# The high frequency of manic symptoms in fibromyalgia does influence the choice of treatment?

**DOI:** 10.1186/1745-0179-2-36

**Published:** 2006-12-19

**Authors:** Mauro Giovanni Carta, Claudia Cardia, Francesca Mannu, Gesuina Intilla, Maria Carolina Hardoy, C Anedda, V Ruggero, D Fornasier, Enrico Cacace

**Affiliations:** 1 Psychiatry, Department of Public Health, University of Cagliari, Italy; 2 Rheumatology, Department of Internal Medicine, University of Cagliari, Italy

## Abstract

**Background:**

Mood disorders were found associated with fibromyalgia (FM) and clinical studies have revealed the efficacy of antidepressant drugs in the treatment of FM. However no specific instruments to identify manic symptoms were used.

**Objectives:**

To assess the frequency of anxiety and mood disorders (particularly bipolar disorders and manic symptoms) in a consecutive sample of women affected by FM using standardized diagnostic tools and to compare the prevalence of these disorders with that observed in a sample of healthy controls from the general population.

**Methods:**

Cases: consecutive series of women (N = 37, mean age 50.1 ± 21.0) attending a Rheumatology outpatient Unit at the University of Cagliari. Controls: 148 women, drawn from the data bank of an epidemiological study matched for sex and age with controls according to a randomisation "after blocks" method. The Italian version of the Composite International Diagnostic Interview Simplified were carried out by physicians. Psychiatric diagnosis was formulated according to DSM-IV criteria. The Italian version of the Mood Disorder Questionnaire (MDQ) was administered to identify manic symptoms and bipolar disorders. Diagnosis of FM were carried out by rheumatologist according to the criteria of American College of Rheumatology.

**Results:**

Subjects with FM showed a higher comorbidity with Generalised Anxiety Disorder, Panic Disorder and Major Depressive Disorder than controls. The study showed a high frequency of manic symptoms (MDQ positive) in the sample of fibromyalgic patients (59%), approximately double that found in the control sample (P < 0.001).

**Discussion:**

Clinical studies have shown the efficacy of antidepressants, especially tricyclic antidepressants, in the treatment of FM. The clinical difficulty in identifying hypomanic episodes is well known particularly where previous and not present episodes are concerned as in depressive patients. These data would suggest further studies on the subject are needed and more caution also in prescribing antidepressants in a population apparently at high risk for bipolar disorders.

## Background

Several studies, reported high frequency of mood disorders in subjects affected by fibromyalgia (FM) [1,2] and short term clinical studies have revealed the efficacy of antidepressant drugs in the treatment of FM [3]. However, no specific instruments to identify manic symptoms were used in these studies. Bipolar Disorders (BD) are renowned to be hard to distinguished by Major Depressive Disorders in research studies when using "traditional" psychiatric diagnostic tools such as interviews carried out by lay interviewer and not by clinicians [4].

The cause of the close association with mood disorders and the efficacy of antidepressants in FM patients is not yet clear. Specific polymorphisms of genes codifying for serotonin transporters and the catechol-O-methyltransferase enzyme which inactivates catecholamines, neurotransmitters implicated in the pathogenesis of affective disorders, have been associated with FM [5]. It has however been acknowledged that the clinical picture of FM often overlaps with those of the Chronic Fatigue Syndrome and Irritable Bowel Syndrome: response to stress is considered to play a crucial role in the pathogenesis of these disorders [6,7]. But the efficacy of antidepressants in this group of disorders may be related to the analgesic effect of these drugs rather that their antidepressant effect and appears strongest in agents with mixed-receptor or predominantly noradrenergic activity, rather than serotoninergic activity [8]

The lack of specific information as to the nature and genesis of FM-associated affective disorders is clinically relevant.

A hypothetical risk of bipolar disorder in fibromyalgic patients has recently been suggested in observational studies [9]; should this hypothesis be confirmed, particular caution would be required in the use of antidepressant drugs. In the course of bipolar disorders, antidepressant drugs are particularly indicated in the case of depressive episodes whilst the use of these drugs alone (without the concomitant use of mood stabilizers) should be avoided due to the serious risk of manic switching and to induce rapid cycling in the long term [10].

## Objectives

The aim of this study is to assess the frequency of anxiety and mood disorders in a consecutive sample of women affected by FM using standardized diagnostic tools administered by clinicians and to compare the prevalence of these disorders with that observed in a sample of healthy controls from the general population. The study also aims to evaluate the presence of manic and hypomanic symptoms among the fibromyalgic patients by means of specific tools such as the Mood Disorder Questionnaire [11].

A comparison of familiarity for mood disorders will be performed between cases and healthy controls.

## Methods

The sample of cases was made up of a consecutive series of women attending the Psychiatric Consultancy Service in the Rheumatology Department of the University of Cagliari.

The case sample included 37 women aged 25 to 72 years, mean age 50,1 ± 21,0 years, whilst controls were drawn from the data bank of an epidemiological study performed on the general Sardinian population [12].

The control group included 148 women and was arranged according to a randomisation "after blocks" method: for each case a "cell" including all control subjects of the same sex and age (± 1 year) as the patients was set up.

Four controls were obtained at random from each cell (4 controls for each case).

Each control was contacted and reassessed (approximately 5 years after the epidemiological survey), 15 women (10.1%) refused to take part in the study or could not be traced. All refusals were substituted by extracting a new proband at random from the same cell as the subject who had refused.

### Clinical assessment and statistical analysis

The Italian version of the Composite International Diagnostic Interview Simplified [13] was used to assess the presence of anxiety and depressive disorders and to verify familiarity for mood disorders among both cases and controls. Interviews were carried out by physicians who had been working in the field of Psychiatry for at least two years. Psychiatric diagnosis was formulated according to DSM-IV criteria.

The Italian version of the Mood Disorder Questionnaire [14] was administered to identify manic symptoms and bipolar disorder. This is a self-administered test which establishes fairly reliably whether an individual has manifested a manic or hypomanic syndrome during his life. The test is made up of 13 closed questions (yes/no answers) and was elaborated bearing in mind the criteria laid down by DSM-IV and by clinical experience.

An excellent degree of sensitivity and specificity of the instrument has been demonstrated by the preliminary validation stages of the Italian version [14] in a sample of subjects attending the Psychiatric Unit of the University for routine psychiatric assessment requested by medical wards.

The test may therefore prove useful in screening for type I and II bipolar disorders in the sample studied here.

The possible presence of somatic disorders among controls, indicating the absence of FM, was evaluated by means of a questionnaire examining physical wellbeing used previously in the epidemiological survey.

The major inclusion criteria for FM were the American College of Rheumatology (ACR) 1990 classification criteria for fibromyalgia [15]: e.g. widespread pain for at least 3 months (pain in the left side of the body, plus right side of the body, plus pain above the waist, plus pain below the waist, plus axial pain; axial pain includes pain in the cervical spine, or thoracic pain, or pain in the low back or anterior chest wall) and the presence of 11 tender points among 18 specified sites. Pressure of 4 kg/cm (enough to whiten the examiner's fingernail) should be applied to each point for a few seconds. Exclusion criteria were concomitant rheumatic diseases. 40 healthy females (aged 27–72, mean age 47.89 + 9.76) were also studied. All FM patients were examined by an experienced rheumatologist. The pain pressure threshold measurements of patients with FM were performed using a mechanical algometer. Pain threshold was defined as the amount of pressure adequate to induce sensation of discomfort, and the subjects were informed that the aim was not to determine pain tolerance. 18 tender points accepted by the ACR for FM were evaluated. All the measurements and tests were carried out by the same doctor throughout the study.

Statistical analysis was performed by assessing the frequency of anxiety and depressive disorders among both cases and controls. The measure of association is expressed as "Odd Ratio" (OR). Confidence intervals of OR were calculated using the simplified method of Miettinen [16]

## Results

Table 1 shows the comparison of frequency in lifetime psychiatric diagnosis between FM patients and controls concerning Generalised Anxiety Disorder, Panic Disorder and Major Depressive Episodes. Subjects with FM showed an higher comorbidity with Generalized Anxiety Disorder, Panic Disorder and Major Depressive Disorder than controls. 19 FM patients presented comorbidity with an anxiety or depressive disorder (51.35%); in 9 FM patients no psychiatric disorders were found (24.32%); 11 controls presented comorbidity with both anxiety and depressive disorders (7.43%, P < 0.001), no psychiatric disorders were found in 91 controls (61.48%, P < 0.001).

**Table 1:** Frequency of psychiatric disorders in patients with fibromyalgia and controls

	Generalized Anxiety Disorder	Social Phobia	Panic Disorder	Major Depressive Disorder	MDQ+ (4 or more symptom + impairment)
CASES	15 (40.5%)	3 (8.11%)	10 (27.1%)	23 (62.16%)	22 (59.4%)
CONTROLS	23 (15.54%)	4 (2.70%)	5 (3.38%)	20 (13.51%)	44 (28.0%)
OR	3.7	3.2	16.8		3.47
X2	9.89	1.12	28.8		10.1
P	0,002	0.289	< 0,001	< 0,001	<0.001

The frequency of bipolar disorders was calculated using a threshold value of 6 as this was the cut-off frequently used in international epidemiological studies: 19 positives (29.7%) were identified among subjects with fibromyalgia compared to 20 in the controls (13.5%, χ^2 ^= 4.48, OR = 2.7, P = 0.034). If we assume as cut-off 4 or more symptoms + impairment as used in Italian studies and as recommended for investigations during the validation stages of the Italian version [14] 22 positive cases (59.4%) were identified among subjects with fibromyalgia compared to 44 (28.0%) in the controls (χ^2 ^= 10.1, OR = 3.5, P = 0.001).

Table 2 shows the comparison between these results and the most recent studies carried out in different settings. Comparison with the US community survey [17] was carried out recalculating the frequencies using the lower comparable cut-off.

**Table 2:** Frequency of MDQ positiveness in recent clinical and epidemiological studies

Study	Year	% Positiveness	Setting
Carta et al. Cagliari (Italy)	2006	59.4 (29.7*)	Fibromyalgic patients
Hardoy et al. Cagliari (Italy)	2005	34.4	Psychiatric patients
Hirshfeld et al. US	2003	3.7*	Community
Mangelli et al. Bologna (Italy)	2005	17.7	Community

*> 6 symptoms i.e. moderate – severe impairment

A familiarity for depressive disorders of 40.54% was found in the FM patients compared to 20.27% in the controls (P < 0.001).

## Discussion and conclusion

The data obtained by us confirmed, by means of a standardised international method, what had already appeared in literature regarding the association between fibromyalgia and anxiety and mood disorders.

A multi-centre study carried out by Epstein et al. [2] showed that anxiety levels seemed correlated in a significant manner to compromised functional ability found in patients with fibromyalgia and noted an important association between depression and fibromyalgia; Cohen H. et al. [18] highlighted the presence of overlap between fibromyalgia and post-traumatic disorders. These authors maintain that common anxiety and depressive disorders are associated with FM and could be independent of the principle symptoms of rheumatic illness and that they could however be correlated to the negative consequences of the effects of the symptoms on quality of life and social function.

However, the close familiarity that emerged from our studies, confirming previous findings [19], cannot exclude the hypothesis of a common biological vulnerability [19].

The most important datum, regarding clinical implications, reported to date, is the high frequency of positive MDQ in the sample of fibromyalgic patients. This frequency is approximately double that found in the control sample and higher than in studies carried out on the general Italian population [20] and in an US community survey [17] the latter comparison, if we re-calculated the prevalence assuming a comparable cut-off (> 6 symptoms i.e. moderate – severe impairment) (see table 2). The frequency of MDQ is even higher to that found in case series of patients attending a public psychiatric service in the same Italian town [14].

The evidence of highly frequent panic and generalised anxiety disorders in the sample of fibromyalgic patients confirms indirectly the hypothesis of the risk of bipolarity. It is known that patients affected by bipolar disorders have a high risk of anxiety disorders, even higher than that of patients affected by major depression [21].

Recent clinical studies have shown the efficacy of anti-depressants, especially tricyclic antidepressants, in the treatment of FM [3]. Their use has consequently been extended to other non psychiatric settings. The clinical difficulty in identifying hypomanic episodes is well known particularly where previous and not present episodes are concerned as in depressive patients [4]. These data would suggest further studies on the subject are needed and more caution also in prescribing antidepressants in a population apparently at high risk for bipolar disorders.

Furthermore, it must considered that in the subjects accepted for clinical studies on depressive disorder in FM, psychiatric diagnosis was carried out by a psychiatrist using proven methodologies, this can render the samples very diverse from cases found in current rheumatological practice, also with regards to the frequency of bipolar depression and to the consequent risk of inducing mania with antidepressants.

Furthermore, clinical trials on the use of anti-depressants in FM are very short and possible negative effects (in the order of onset of maniacal symptoms, mixed and/or induction of rapid cycles) should be evaluated over the medium term considering that most of the patients have some ongoing affective disorder on entering the trials.

Considering that the efficacy obtained in clinical studies by some psychotherapic treatments has been shown to be virtually identical to that when using antidepressants in FM [22,23], should our data be confirmed, then the consequence would be that psychotherapic treatments of proven efficacy in FM should be considered first choice compared to antidepressants.

## Limits

The study used healthy controls, when in most studies the controls for FM patients are patients with Rheumatoid Arthritis. In fact, it's obvious that anxiety/depressive symptoms/disorders are much more common among non-healthy than among healthy people. But this discrepancy may be just due to dificulties in cope with a disease (such as FM). Nevertheless the main result of this study is the high frequency of MDQ positives in the sample of fibromyalgic patients suggesting high rates of bipolar disorders. This finding was never observed in other rheumatic disorders. Future studies need to confirm if the association with bipolar disorders is specific for fibromyalgia or it is common in other rheumatic disorders.
